# Investigating Fall-Related Factors in Community-Dwelling Older Women Through Structural Equation Modeling Analysis

**DOI:** 10.3390/ijerph22060906

**Published:** 2025-06-06

**Authors:** Riskah Nur’amalia, Mayumi Kato, Masami Yokogawa, Yoshimi Taniguchi, Andi Masyitha Irwan

**Affiliations:** 1 Doctoral Course of Graduate School of Medical Sciences, Kanazawa University, Kanazawa 9200942, Japan; 2 Department of Physiotherapy, Hasanuddin University, Makassar 90245, Indonesia; 3 Faculty of Health Sciences, Institute of Medical, Pharmaceutical and Health Sciences, Kanazawa University, Kanazawa 9200942, Japan; mykato@mhs.mp.kanazawa-u.ac.jp (M.K.); yokogawa@mhs.mp.kanazawa-u.ac.jp (M.Y.); ytani@staff.kanazawa-u.ac.jp (Y.T.); 4 Faculty of Nursing, Hasanuddin University, Makassar 90245, Indonesia; citha_ners@med.unhas.ac.id

**Keywords:** fall, gait efficacy, older women, physical activity, structural equation modeling

## Abstract

Falls are more prevalent in older women than in older men; however, few structural equation analysis studies have focused on the factors contributing to falls in this population, particularly in Asian regions such as Indonesia. This study aimed to investigate the direct and indirect associations among fall-related factors in community-dwelling older women. This cross-sectional study enrolled 90 community-dwelling older women aged ≥ 60 years from August to September 2023. Data collection included structured questionnaires on fall incidence as a sociodemographic variable, fear of falling, and gait efficacy, as well as physical measurements of physical function and the amount of physical activity. Structural equation modeling was used to test the hypothesized pathways among the variables. The results showed that physical function (β = 0.233, *p* = 0.02), gait efficacy (β = −0.318, *p* = 0.001), and amount of physical activity (β = −0.243, *p* = 0.009) were directly associated with fall incidence. Physical function (β = 0.152) and fear of falling (β = 0.183) were indirectly associated with fall incidence through the mediation of gait efficacy and the amount of physical activity. Furthermore, the amount of physical activity was directly associated with high physical function (β = −0.236, *p* = 0.038). These findings suggest that, in addition to improving physical function and activity levels, older women require psychometric interventions to prevent falls.

## 1. Introduction

Falls in community-dwelling older adults are a major public health concern worldwide, with one in three older adults experiencing a fall each year, leading to a loss of independence, decreased quality of life, an economic burden, and even death [1,2,3]. Fall is defined as an unexpected event where an individual ends up on the ground, floor, or a lower level [3]. Compared with older men, older women are more likely to experience falls and fall-related injuries in the community, with 40% of falls in older women resulting in injury and 30% requiring medical attention [3,4,5]. The high prevalence, severe consequences, and multifactorial nature of falls in community-dwelling older women highlight the critical need for continued research in this area to develop and implement effective prevention strategies.

Several factors contribute to higher fall rates in older women than in older men, including physiological differences such as women’s experience of more bone mineral density loss and higher osteoporosis due to menopause [6]. Aging is also associated with increased inactivity. Notably, one in two women compared with one in three men do not engage in regular physical activity [7]. Moreover, older women consider themselves as caregivers with a responsibility to care for spouses, children, or grandchildren and perform household roles, which can hinder their engagement in regular physical exercise and increase the risk of falls [8]. In the psychological field, fear of falling is reported as a risk factor for falls [9]. Although discrepancies in physiological, psychological, and lifestyle factors have been individually studied, research examining the interactions among these factors to influence fall incidence is still limited.

Structural equation modeling (SEM) has been widely used in clinical contexts to investigate a variety of health-related outcomes. Dai and colleagues [10] used SEM to clarify the interrelationships between physical function, impaired mobility, and falls. Physical function, including balance, strength, endurance, and flexibility, is directly associated with impaired mobility and falls. Similarly, SEM was also used to analyze the factors associated with fear of falling in community-dwelling older adults. The findings revealed that being female, having worse physical performance, and having a higher number of self-reported morbidities were directly associated with greater fear of falling among community-dwelling older adults [11]. The study showed that fear of falling was not directly associated with falls, which contrasts the findings of other studies suggesting a direct relationship between fear of falling and fall incidence. This inconsistency highlights a critical gap in the literature, emphasizing the need for further investigation to clarify the role of fear of falling in fall incidence, particularly among older women. This approach also highlights the potential for integrating SEM with objective measures to capture a more comprehensive understanding of the complex pathways influencing mobility and falls in this population.

Integrating both physical and psychological factors into a unified model may enhance the quality of research on fall prevention and provide a direction for future studies to examine the relationships among the factors in detail. Physical function is an objectively observable systemic mobile function of the body [12]. Its deterioration is associated with aging, such as muscle weakness, balance problems, and gait instability, which increases fall risk [13]. Psychological factors such as fear of falling also contribute to fall risk [14]. Fear of falling is defined as a persistent concern about falling, leading to an individual’s avoidance of activities they can perform [15]. This psychological barrier compromises people’s confidence in their ability to ambulate safely, leading to the avoidance of activities, further decline in physical function, and the onset of disuse syndrome. A higher prevalence of fear of falling and other psychological concerns related to falls is commonly observed in women compared to men, affecting women’s physical activity levels and function [16]. Gait efficacy, a related psychological construct, refers to the confidence and self-belief of individuals in their ability to walk safely and avoid falls, which contributes to mitigating fall risk [17]. This concept aligns closely with Bandura’s Self-Efficacy Theory, which posits that individuals with higher self-efficacy are more likely to engage in activities despite potential obstacles. In comparison, those with lower self-efficacy tend to avoid activities due to perceived risks. According to Bandura (1997) [18], self-efficacy is shaped from four main sources: mastery experiences, vicarious learning, social persuasion, and physiological states. These sources directly influence gait efficacy, as older adults who have successfully navigated physical challenges, observed peers overcoming similar difficulties, received encouragement from health professionals, or maintained better physical health tend to report higher gait efficacy. This, in turn, promotes greater participation in physical activities and reduces the risk of functional decline. Furthermore, gait efficacy mediates the association between physical activity, physical function, and functional limitation in older adults [19,20]. Therefore, the interplay between physical function, physical activity, fear of falling, and gait efficacy is critical for understanding its relationship with falls in this population.

The originality of this research lies in several aspects. First, while previous research has focused on examining physical or psychological factors independently, this is the first study to use SEM in a single framework. Some studies relied on self-reported measures of physical activity; however, this study used objective and reliable measures of activity to monitor step count and various walking tests. These objective measurements offer more accurate assessments of the amount of physical activity and physical function, reducing the potential bias associated with self-reports. The current research also provides theoretical guidance and practical references for fall prevention in older women. This study aimed to investigate the direct and indirect associations among fall-related factors in community-dwelling older women.

## 2. Materials and Methods

### 2.1. Study Design

**Figure 1:**
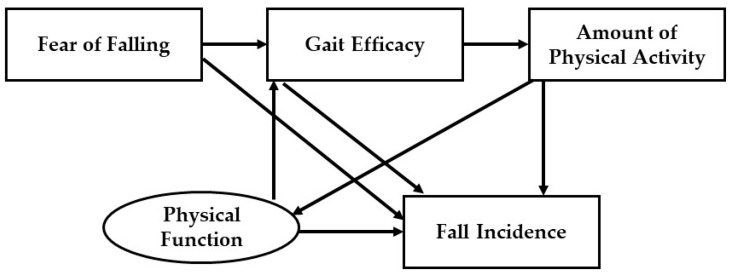
The hypothetical model.

This cross-sectional study used SEM to examine the study’s hypotheses. Figure 1 outlines the study’s hypotheses. H1: Physical function, fear of falling, gait efficacy, and amount of physical activity are directly associated with fall incidence. H2: Fear of falling and physical function mediated by gait efficacy and the amount of physical activity are indirectly associated with fall incidence. H3: The amount of physical activity is directly associated with physical function.

### 2.2. Structural Equation Modeling

In this study, the relationship model (Figure 1) was developed based on a theoretical framework that consists of one main theory (Self-Efficacy Theory) combined with other concepts. The boxes indicate observed variables, and the circles indicate latent variables. The arrows indicate the direction of impact.

### 2.3. Participants

This study was conducted in Gowa Regency, South Sulawesi, Indonesia, from August to September 2023. All community-dwelling older women aged ≥ 60 years old in this area, who could walk independently, and who did not have hearing and visual impairments, were recruited and included in this study, resulting in 90 participants. Individuals with severe cognitive impairment, osteoarthritis, and cardiovascular diseases were excluded. The appropriate number of participants required for SEM is 80–100 [21]. The required sample size was also calculated using G*power 3.1 analysis [22], setting a medium effect size of 0.15, an α level of 0.05, and a power of 0.8. The independent variables in this study’s hypothesis were four, resulting in an estimated sample size of 85; therefore, including 90 participants was sufficient for the study’s analysis. The effect sizes used in the G*Power software were comparable to those obtained in the SPSS analysis. In the correlation analysis test, the sample size met the criterion for a medium effect size, and the effect power was high. Therefore, the results of this study are considered valid. This study’s design was based on recommendations from the Strengthening the Reporting of Observational Studies in Epidemiology protocol [23]. This study was approved by the Medical Ethics Review Committee of Kanazawa University (approval number: 111094-1). This research complied with the “World Medical Association Declaration of Helsinki” and the “Ethical Guidelines for Life Science and Medical Research Involving Human Subjects” (provided by Ministry of Education, Culture, Sports, Science and Technology; Ministry of Health, Labor and Welfare; and Ministry of Economy, Trade and Industry of Japan). Written informed consent was obtained from all the participants.

### 2.4. Measurements

Sociodemographic variables included age, education, living status, toilet use, stair use, working status, health status, and fall incidence. Data on the incidence of falls were collected by asking the participants to answer yes/no to “Have you ever fallen in the past year?”. Respondents who had experienced a fall were considered fallers. Fall incidence was included as a covariate in the final model to control for its potential influence on the outcomes of interest, allowing for a clearer examination of the relationships between other variables.

Fear of falling was examined using the Short Fall Efficacy Scale International (Short FES-I), which measures fear of falling during daily activities. This scale consists of seven items related to concerns about falling with four response options (from 1 = not concerned at all to 4 = very concerned). The total score is calculated as the sum of the scores, which varies from 7 to 28, with lower values representing the absence of concerns about falling and higher scores representing greater concerns about falling. The scale demonstrated excellent internal consistency with a Cronbach’s alpha value of = 0.87 [24].

Gait efficacy was measured using the Modified Gait Efficacy Scale. This scale asked questions about the degree of confidence in walking under various conditions and consisted of 10 items, with a response of 1 indicating no confidence and 10 indicating complete confidence. The total score is calculated as the sum of the scores, which varies from 10 to 100, with a higher total score representing a higher degree of confidence in all tasks. The scale demonstrated excellent internal consistency with a Cronbach’s alpha value of = 0.94 [25].

Physical function was assessed using (1) the Five-Meter Walking Test (5 MWT), in which participants were asked to walk in a straight 5 m line at their usual walking speed (the results were recorded in seconds) [26]; (2) the Zig-Zag Walking Test (ZWT), in which the participants were asked to walk a 3 m walking path with four turns at a marked point at their usual walking speed, and the time was measured and recorded in seconds [27]; (3) and the Timed Up and Go Test (TUGT), in which the participants were asked to stand from the sitting position on the chair, walk along a straight 3 m walkway, turn at a marked point, walk along a straight 3 m walkway, and return to the original chair to sit. The time (seconds) required to complete the test was measured using a standard stopwatch. A chair of a standard height (approximately 40 cm) without wheels was used in this study [28,29]. We asked the participants not to touch the chair when standing. All physical function measurements were repeated twice, and the mean values were analyzed.

The amount of physical activity was measured using the Activity Monitor EZ-064 (Tanita Co., Ltd., Tokyo, Japan), which was used to measure the number of steps taken over 7 days. The average number of steps from days 2 to 6 was calculated for the analysis. The activity meter was 71 mm wide, 33 mm high, and 15 mm deep, and weighed 28 g. The participants wore an activity meter fixed to a belt for 1 week, except when bathing and changing clothes. The accuracy of this activity monitor was tested using a vibration testing machine and found to be within ±5% in counting steps [30]. The manual of this activity monitor is available online at https://www.tanita.co.jp/support/manual/EZ-064 (accessed on 13 March 2023).

### 2.5. Statistical Analysis

Data analyses were performed using SPSS version 29 (IBM Corporation, Armonk, NY, USA) and AMOS version 29 (IBM Corporation). Descriptive statistics were used to describe the sample characteristics and are represented as means ± standard deviations or frequencies (percentages). Bivariate associations between variables were tested, and the independent sample *t*-test, Fisher’s exact test, or Chi-square test was used to determine the significance between variables in fallers and non-fallers. *p*-values < 0.05 were considered statistically significant. Collinearity diagnostics were performed before the hypothetical analyses. A variance inflation factor value < 10 and tolerance value > 0.10, which included the variables in the SEM analyses, indicate no multicollinearity [21]. Variables with a variance inflation factor greater than 10, indicating high multicollinearity, were excluded from the SEM analysis to ensure the reliability of the results.

SEM was conducted to evaluate the hypothesized associations among variables. A two-step approach to SEM was used. First, confirmatory factor analysis (CFA) was conducted to confirm whether the indicators (5 MWT, ZWT, and TUGT) reliably measure the latent variable (physical function). Second, the final model, including physical function, fear of falling, gait efficacy, physical function, and fall incidence variables, was tested. The dependent variable, fall incidence, was a binary variable coded as 0 (no fall) and 1 (fall). The structural equation model was estimated using AMOS with the default maximum likelihood estimation method. Model fit was assessed using the recommended thresholds, including χ^2^/df ≤ 2, the comparative fit index (CFI) > 0.95, the normed fit index (NFI) > 0.95, the goodness-of-fit index (GFI) > 0.95, and the root mean square error of approximation (RMSEA) < 0.08, supporting the validity of the proposed relationships [21,31].

## 3. Results

### 3.1. Participant Characteristics

Table 1 presents the demographic characteristics of the 90 participants. Their mean age was 68.0 years (standard deviation = 6.4). The number of fallers who had reported at least a fall within a year was 19 (21.1%). The mean 5 MWT, ZWT, and TUGT speeds were significantly higher in fallers than in non-fallers (all *p* < 0.001). Furthermore, the mean Modified Gait Efficacy Scale score and mean number of steps were significantly lower in fallers than in non-fallers (both *p* < 0.001). However, no significant difference existed in the Short FES-I scores between fallers and non-fallers.

Age was included as the controlling variable due to its well-established role as a significant predictor of fall incidence and physical function in older adults. Age has been widely documented as a key factor influencing mobility and fall risk, informing its inclusion as a control variable. Although other sociodemographic factors, such as living status and education, were measured, they were not included in the model as control variables.

### 3.2. SEM Examination

**Figure 2:**
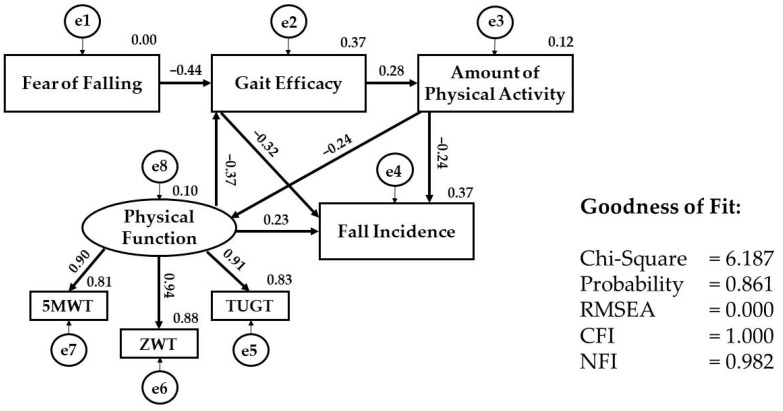
Structural equation modeling analysis. Note: 5 MWT, Five-Meter Walking Test; ZWT, Zig-Zag Walking Test; TUGT, Timed Up and Go Test; RMSEA, root mean square error of approximation; CFI, comparative fit index; NFI, normed fit index.

Figure 2 shows the final model of the SEM analysis. No multicollinearity was observed among the variables. Therefore, the measurement model comprising physical function, fear of falling, gait efficacy, physical activity, and fall incidence in older women was analyzed using SEM. The CFA for the latent variable physical function, measured by the 5 MWT, ZWT, and TUGT, demonstrated a good model fit (CFI = 1.000, GFI = 1.000, NFI = 1.000), with factor loading of 5 MWT, ZWT, and TUGT of 0.901, 0.942, and 0.910, respectively, supporting the adequacy of the measurement model prior to testing the full structural equation model. In the initial model, fear of falling was included as a direct predictor of fall incidence. However, the model fit was not satisfactory, leading to a modification where the pathway between fear of falling and fall incidence was removed. This modification improved the overall model fit, which was subsequently assessed using various fit indices. Fear of falling was found to be significantly associated with other factors such as gait efficacy and physical activity; however, its direct impact on fall incidence was not supported by the model. To obtain an appropriate model, we modified the initial model. The modified model showed an excellent fit, which was represented by the following: χ^2^ = 6.187, df = 11, χ^2^/df = 0.562, probability = 0.861, NFI = 0.982, CFI = 1.000, and RMSEA = 0.000.

Table 2 shows the associations among the variables by regression weight, which exhibited statistically significant direct and indirect estimators. Lower physical function (β = 0.233, *p* = 0.02), gait efficacy (β = −0.318, *p* = 0.001), and amount of physical activity (β = −0.243, *p* = 0.009) had direct associations with fall incidence. Furthermore, lower physical function (β = 0.152) and higher fear of falling (β = 0.183) had indirect associations with fall incidence through the mediations of lower gait efficacy and amount of physical activity. Lastly, a higher amount of physical activity was directly associated with higher physical function (β = −0.236, *p* = 0.038). However, fear of falling only had an indirect effect on fall incidence through its relationship with gait efficacy and the amount of physical activity.

## 4. Discussion

This is the first study to examine the direct and indirect associations between physical function, fear of falling, gait efficacy, amount of physical activity, and fall incidence among community-dwelling older women, using the SEM approach. In this study, all factors, except fear of falling, were directly associated with fall incidence. We also found that physical function and fear of falling were indirectly associated with fall incidence through the mediation of gait efficacy and amount of physical activity. Additionally, the amount of physical activity was directly associated with physical function. These findings contribute to the provision of theoretical guidance on fall prevention among community-dwelling older women.

These direct associations are consistent with the results in previous studies showing that physical function [32,33,34], gait efficacy [35], and amount of physical activity [33,36,37] were associated with fall incidence. As women age, their physical function tends to decline, particularly after 60 years of age [38]. The 5 MWT, ZWT, and TUGT, which assess physical function, incorporate walking tasks that simulate real-life walking environments. The straight pathway in the 5 MWT and multiple turns in the ZWT and TUGT reflect walking during typical daily activities, where both linear movements and turns or changes in direction are made. Considering that turns compared with straight walking require more balance and coordination, the ZWT, with several turns, may be a valid predictor of falls [28]. The TUGT combines the sit-to-stand, walking, turning, and sit-down movements to assess agility and dynamic balance. In this study, the TUGT was also used to identify significant differences between the fallers and non-fallers. The fallers group had significantly higher TUGT time (mean 17.1 ± 5.9 s) compared with that of the non-fallers group (mean 12.8 ± 3.1 s), which is consistent with the established threshold that TUGT time durations greater than 13.5 s reflect increased fall risk [29,39].

Significantly, the findings of this study support the hypothesis that fear of falling and physical function, mediated by gait efficacy and the amount of physical activity, are indirectly associated with fall incidence. We also found that fear of falling was indirectly associated with the amount of physical activity in older women through the mediation of gait efficacy. This finding is consistent with dos Santos and colleagues who found that fear of falling was indirectly associated with physical inactivity [11]. Regarding Bandura’s social learning theory about fall-related self-efficacy, self-efficacy influences behavioral decisions, including the choice to engage in certain activities [40]. When gait efficacy is low, older women may perceive themselves as more vulnerable during activities involving walking, even if they are capable of safely engaging in those activities. Consequently, diminished confidence leads to reduced physical activity and may prevent certain movements or exercises that increase the risk of falls [17,19]. Improving gait efficacy potentially helps to prevent or mitigate disuse syndrome and fall incidence by promoting physical activity and maintaining functional abilities [41]. The findings of this study underscore the direct association between the amount of physical activity and physical function among community-dwelling older women. Consistent with this finding, Fushimi and colleagues found that physical activity is associated with physical function [42]. The use of step count measurements over 5 days to assess the amount of physical activity is considered an objective assessment of a participant’s habitual physical activity and reduces the potential bias associated with self-report measures [43]. Our study’s results showed that the non-fallers group had a significantly higher step count (mean 4237.5 ± 3042.5 steps) than that of the fallers group (mean 1029.2 ± 782.1 steps). The step count data in the non-fallers group are consistent with the step count range that reflects the amount of physical activity in healthy older adults, which is 2000–8000 steps daily [44], supporting the validity of the step count measurement in this population. This finding emphasizes the importance of increasing the physical activity level to maintain physical function in older women.

Older women compared with older men are more engaged in domestic activities, such as managing the household and taking care of children, grandchildren, or other dependent family members. Housework can be physically demanding and hinder women’s ability to participate in regular physical exercise, leading to decreased physical function [45]. In addition, owing to aging, women experience hormonal changes during menopause, as well as certain health conditions, such as osteoporosis and arthritis. These conditions can limit their mobility, contribute to muscle loss, and decrease physical function. This suggests that older women face unique challenges that require specialized interventions. It is necessary to consider strategies that encourage physical activity in ways that align with older women’s daily routines. For example, intervention could focus on incorporating more physical activity into daily life routines through modified household tasks, such as walking or moving around during housework, which may help women maintain functional mobility, maintain muscle strength, and prevent falls. Furthermore, healthcare providers and public health initiatives should emphasize the benefits of regular walking or increasing steps in older women, even when domestic responsibilities limit the time available to engage in such physical activities.

In the hypothesis model, we proposed that fear of falling would be directly associated with fall incidence; however, we found no significant direct association between fear of falling and fall incidence. Fear of falling is indirectly associated with fall incidence through the mediation of gait efficacy and the amount of physical activity. This finding is consistent with that of dos Santos and colleagues who highlighted that fear of falling was indirectly associated with physical inactivity [11]. In contrast, Lavedán and colleagues reported a significant positive association between both variables [9]. One possible explanation for this difference is the variation in study populations. Our study focused exclusively on older women, whereas both dos Santos and Lavedán and their colleagues included mixed-sex samples, potentially introducing different physical and psychological characteristics that could influence the direct impact of fear of falling. Additionally, we excluded older adults with disabilities, which may explain why fear of falling was not directly associated with fall incidence in our study. This distinction could contribute to the observed differences between our findings and those in the existing literature. In this study, we suggested the indirect association between fear of falling and fall incidence. Specifically, fear of falling negatively influences gait efficacy, which in turn reduces physical activity. Both lower gait efficacy and reduced physical activity may contribute to an increased fall incidence in older women. Women’s muscle mass, particularly in the year of menopause, declines faster than that observed in men. Therefore, older women need special support to engage in regular exercise or improve physical activity levels, which may alleviate their fear of falling. Furthermore, the recognition of physical decline increases the fear of falling [46]. Enhancing confidence in walking abilities (i.e., increasing gait efficacy) and encouraging more physical activity may reduce the negative impact of fear of falling and lower the incidence of falls in community-dwelling older adults.

In the present study, SEM was selected to examine the complex relationships between physical function, fear of falling, and fall incidence. SEM allows for the inclusion of latent variables, such as physical function, and the testing of both direct and indirect effects. This makes SEM particularly suitable for understanding the multifactorial nature of fall risk in older adults. It allows for a simultaneous analysis of multiple relationships in a single model. In contrast, logistic regression analysis would require separate models to explore these indirect effects, limiting the ability to capture complex interdependencies [21]. Moreover, SEM helps to control for measurement error in latent variables, such as physical function, which cannot be directly observed and is typically measured through multiple indicators (e.g., tests of walking speed, balance, and strength). Logistic regression analysis cannot account for such measurement error, potentially leading to biased results.

Overall, the findings from our model suggest a cycle between physical function, gait efficacy, and the amount of physical activity, which potentially affects the incidence of falls. A negative effect on certain factors, such as a decline in physical function due to aging, leading to decreased gait efficacy and a reduced amount of physical activity, could create a cyclic effect on the occurrence of falls. During the cycle, as each factor continues to worsen, it could exacerbate the next factor. Low physical function can lead to a reduced amount of physical activity by decreasing gait efficacy. Conversely, a low amount of physical activity can result in decreased physical function. As the amount of physical activity decreases owing to low gait efficacy, muscle strength and balance may further deteriorate, leading to even lower confidence in walking abilities and creating a negative cycle of reduced activity, which potentially becomes a factor of fall incidence [47,48]. Low gait efficacy may also create psychological barriers to engaging in physical activity because older women may feel less motivated or capable of participating in exercise programs or daily activities, which contributes to the development of disuse syndrome [49]. This situation creates a cycle where each factor reinforces the other factors. Our study showed that each factor, except for the fear of falling, directly affected fall incidence. Therefore, there is an urgent need to develop effective strategies to minimize the incidence of falls that focus on older women by targeting physical function, gait efficacy, and the amount of physical activity.

The study findings highlight the need to address not only improvements in physical function and physical activity but also address gait efficacy and fear of falling in fall prevention programs for older women. Healthcare providers should provide personalized physical activity programs based on the status of the above-mentioned factors and consider referring older women to physical therapy or exercise classes using Geragogy, which focuses on fall prevention. Effective instructions for older women involve using various strategies, such as using interactive instruction, vivid visual (booklet or poster) and auditory cues, facilitating discussions, and providing practice opportunities to stimulate interest and maintain motivation [50]. It is also essential to incorporate discussions about fear of falling into regular consultations to help address psychological barriers. Additionally, healthcare providers should encourage family members to actively participate in household activities, cooperate with older women to reduce domestic workload, and promote more engagement in exercise. These comprehensive programs might help older women to meet their unique needs, promoting both physical and psychological aspects in falls prevention.

This study has some limitations that should be addressed in future studies. First, the participants were recruited from a single geographical location, which may limit the generalizability of the results. Second, the cross-sectional nature of the study precludes definitive statements about cause-and-effect relationships. Future research should incorporate longitudinal designs to infer causality. Third, although age was defined and included as a confounding factor in the analysis, other potential confounders were not identified or controlled for. This could impact the interpretation of the results. Future research that identifies and accounts for additional confounding factors that may influence fall-related outcomes should be conducted. We acknowledge that potential bias may arise from self-reported falls. However, in this study, with a fall rate of 21.1%, the findings are considered to be a valid representation of the older adult population. The fall rate in this study is consistent with the global fall rates for older adults, which is 20–30%, depending on the specific region and age group [3]. In addition, this study performed SEM using AMOS with the default maximum likelihood method. As AMOS does not support the logit or probit link functions for modeling categorical endogenous variables, there is a potential for biased estimates when binary outcomes are included. Future research should consider using software that accommodates categorical outcomes, such as Mplus, when estimating SEM. Finally, the model used in this study was constructed based on the study literature. Therefore, the model may be limited in its ability to explain the complex interplay of fall-related factors in community-dwelling older women. The generalizability of these study findings to older men should be assessed in future studies.

## 5. Conclusions

Our findings highlight the interconnected psychological and physical factors that contribute to fall risk in older women. Physical function, gait efficacy, and amount of physical activity were directly associated with fall incidence. Physical function and fear of falling, mediated by gait efficacy and amount of physical activity, were associated with fall incidence. Furthermore, physical function was directly associated with physical activity. These findings suggest the need to consider these factors when designing an effective fall prevention program for older women. Specifically, educational programs using Geragogy and awareness-empowering initiatives should be promoted to address these factors and enhance fall prevention outcomes.

## Figures and Tables

**Table 1:** Characteristics of fallers and non-fallers.

Characteristics		Total (N = 90) Mean ± SD/Number (%)	Fallers (N = 19) Mean ± SD/Number (%)	Non-Fallers (N = 71) Mean ± SD/Number (%)	*p*-Value
Age (years)		68.0 ± 6.4	67.6 ± 5.1	68.2 ± 6.7	0.730 ^a^
Educational Background	Less than middle school	71 (78.9)	16 (84.2)	55 (77.5)	0.753 ^b^
	Middle and high school	19 (21.1)	3 (15.8)	16 (22.5)	
Living Status	Living alone	10 (11.1)	3 (15.8)	7 (9.9)	0.435 ^b^
	Living with spouse/children, relatives	80 (88.9)	16 (84.2)	64 (90.1)	
Toilet Use	Sit	24 (26.7)	6 (31.6)	18 (25.4)	0.586 ^c^
	Squat	66 (73.3)	13 (68.4)	53 (74.6)	
Stair Use	Yes	30 (33.3)	6 (31.6)	24 (33.8)	0.855 ^c^
	No	60 (66.7)	13 (68.4)	47 (66.2)	
Working Status	Yes	22 (24.4)	5 (26.3)	17 (23.9)	1.000 ^b^
	No	68 (75.6)	14 (73.7)	54 (76.1)	
Health Status	Good	65 (72.2)	13 (68.4)	52 (73.2)	0.677 ^c^
	Not good	25 (27.8)	6 (31.6)	19 (26.8)	
Physical Function	5 MWT usual walk (second)	8.2 ± 2.4	10.2 ± 3.3	7.7 ± 1.8	<0.001 ^a^
	ZWT average 1st and 2nd walk (second)	13.3 ± 4.7	17.1 ± 6.9	12.3 ± 3.3	<0.001 ^a^
	TUGT average 1st and 2nd walk (second)	13.7 ± 4.2	17.1 ± 5.9	12.8 ± 3.1	<0.001 ^a^
Fear of Falling	Short FES I	11.4 ± 4.3	12.0 ± 4.8	11.3 ± 4.1	0.517 ^a^
Gait Efficacy	mGES	84.7 ± 15.6	69.4 ± 21.3	88.9 ± 10.6	<0.001 ^a^
Amount of Physical Activity	Number of steps (steps/day)	3560.2 ± 3022.9	1029.2 ± 782.1	4237.5 ± 3042.5	<0.001 ^a^

Note: 5 MWT, Five-Meter Walking Test; TUGT, Timed Up and Go Test; ZWT, Zig-Zag Walking Test; mGES, Modified Gait Efficacy Scale; Short FES I, Short Fall Efficacy Scale International; SD, standard deviation, ^a^: Independent sample *t*-test; ^b^: Fisher’s exact test; ^c^: Chi-square test.

**Table 2:** Regression weights.

Path	β (Estimator)	S.E.	*p*-Value
Fear of falling → Gait efficacy	−0.444	0.308	<0.001
Gait efficacy → Fall incidence	−0.318	0.003	0.001
Amount of Physical activity → Fall incidence	−0.243	0.000	0.009
Physical function→ Fall incidence	0.233	0.011	0.020
Gait efficacy → Amount of Physical activity	0.283	20.700	0.008
Physical function → Gait efficacy	−0.369	0.370	<0.001
Physical activity → Physical function	−0.236	0.000	0.038

Note: β = standardized coefficient, S.E. = standard error.

## Data Availability

The data presented in this study are not available due to ethical restrictions.

## Abbreviations

The following abbreviations are used in this manuscript:

SEM	Structural equation modeling
Short FES-I	Short Fall Efficacy Scale International
5MWT	Five-Meter Walking Test
TUGT	Timed Up and Go Test
ZWT	Zig-Zag Walking Test
CFA	Confirmatory factor analysis
CFI	Comparative fit index
NFI	Normed fit index
GFI	Goodness-of-fit index
RMSEA	Root mean square error of approximation
